# A Label‐Free Multitechnique Approach to Characterize the Interaction of Bioactive Compounds with Biomimetic Interfaces

**DOI:** 10.1002/smsc.202300271

**Published:** 2024-03-01

**Authors:** Eduarda Fernandes, Rui R. Costa, Raúl Machado, Rui L. Reis, Iva Pashkuleva, Marlene Lúcio

**Affiliations:** ^1^ CF‐UM‐UP Centro de Física das Universidades do Minho e Porto Universidade do Minho 4710‐057 Braga Portugal; ^2^ 3B's Research Group, I3Bs – Research Institute on Biomaterials, Biodegradables and Biomimetics University of Minho, Headquarters of the European Institute of Excellence on Tissue Engineering and Regenerative Medicine, AvePark, Parque de Ciência e Tecnologia, and ICVS/3B's, PT Government Associate Laboratory 4805‐017 Braga/Guimarães Portugal; ^3^ CBMA Centro de Biologia Molecular e Ambiental/Aquatic Research Network (ARNET) Associate Laboratory, and IB‐S, Institute of Science and Innovation for Bio‐Sustainability Universidade do Minho Campus de Gualtar 4710‐057 Braga Portugal; ^4^ CF‐UM‐UP, Centro de Física das Universidades do Minho e Porto, and CBMA Centro de Biologia Molecular e Ambiental Universidade do Minho 4710‐057 Braga Portugal

**Keywords:** biomimetic models, label‐free interaction characterizations, quartz‐crystal microbalances, small and wide‐angle X‐Ray scattering, supported lipid bilayers, surface plasmon resonances

## Abstract

Extensive research has been conducted on biomimetic interfaces mimicking the complex and diverse microenvironment of cell membranes to gain insights into bioactive compound interactions and membrane biophysics modulation. The present study proposes an innovative approach that combines five prospective label‐free methodologies (derivative spectroscopy, synchrotron small‐ and wide‐angle X‐Ray scattering, attenuated total reflection–Fourier‐transform infrared spectroscopy, quartz‐crystal microbalance with dissipation, and surface plasmon resonance) to showcase their synergistic capabilities and complementarity in investigating drug–membrane interactions. This multitechnique approach combines the real‐time monitoring of the adsorption process under continuous flow conditions with the steady‐state perspective of this process. As a proof of concept, the interaction of three bioactive compounds (caffeine, testosterone, and diclofenac) with two biomimetic membrane interfaces (multistacked lipid bilayers and supported lipid bilayers) mimicking the more ordered lipid transient phases, with and without cholesterol (*l*_o_ and *s*_o_), that are responsible for a variety of membrane‐associated biological activities, is investigated. The biophysical effects of the bioactives are discussed using complementary data from real‐time and steady‐state experiments, including membrane adsorption and distribution, predicted location, and induced changes in order and fluidity, encompassing bilayer thickness, hydration, and area per lipid molecule.

## Graphical abstract

This work introduces a label‐free multitechnique approach to explore the biophysics underlying the interactions between bioactives and biomimetic models of the often‐overlooked solid‐ and liquid‐ordered phases. The combination of real‐time continuous flow with equilibrium steadystate techniques was successful in providing comprehensive molecular insight into drug‐membrane interactions while overcoming the limitations of conventional fluorescence‐based techniques.

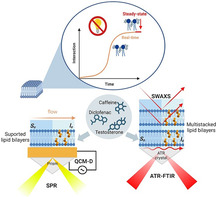

## 1 Introduction

Cell membranes consist of sheet‐like assemblies of thousands of amphiphilic lipid molecules held together by hydrophobic interactions between lipid acyl chains.^[^ ^1^ ^]^ Nearly half of all known drug targets and almost all key drug‐metabolizing enzymes are found within cells.^[^ ^2^ ^]^ Therefore, for administrated drugs and other bioactive compounds to reach their targets, they must traverse the cellular membrane via drug transport mechanisms. The majority of these mechanisms is regulated by the ability of the active agents to interact and passively diffuse through the lipid membrane matrix. In this regard, the lipophilicity of bioactive compounds (i.e., their hydrophilic/lipophilic balance) governs their distribution at the cellular membrane. Consequently, this distribution influences their pharmacokinetic profile, which is determinant for their bioactivity and bioaccumulation.[^2^, ^3^]

Biomimetic interfacial systems that mimic the lipid matrix of cell membranes have been widely developed and used to study the lipophilicity profile of bioactives.^[^ ^4^ ^]^ These biomimetic membrane models are an excellent tool for screening and evaluating new drugs or bioactives,^[^ ^1^, ^2^, ^3^, ^5^ ^]^ as well as for a better understanding of the molecular mechanisms underlying the therapeutic and toxic effects of already clinically established drugs^[^ ^6^ ^]^ and widely used bioactives.^[^ ^7^ ^]^ Compared to more complex models used in cellular in vitro studies, biomimetic membrane models offer several advantages: they enable studies under controlled and adjustable conditions, and their simplicity allows for straightforward characterization of interactions and processes at the molecular level.^[^ ^1^, ^2^, ^8^ ^]^ In contrast to other simpler in vitro models, such as octanol:water binary systems or in silico computational models, biomimetic membrane models can mimic pH‐dependent ionization states, electrostatic interactions, hydrogen bonding to polar headgroups, and ionic strength. This level of accuracy is unattainable in octanol:water systems and is rarely considered in current simulation studies.[^6^, ^9^]

Phosphatidylcholine, including its palmitoyl derivative (e.g., dipalmitoylphosphatidylcholine, DPPC), represents a major lipid component of cell membranes. Due to its ability to mimic the physical–chemical and biological properties of cellular membranes, DPPC is extensively used for the assembly of biomimetic membrane models.[^2^, ^6^, ^10^] When DPPC is dispersed in water, it spontaneously forms multilamellar vesicles, which possess several characteristics resembling cellular membranes. These include the barrier function and the ability to be permeable through passive diffusion across the bilayer.^[^ ^11^ ^]^ Another important feature is the ability to mimic the biophysical states of the cellular membrane, characterized by the lateral organization and molecular order of lipid molecules. Single‐component phospholipid bilayers of DPPC are frequently used to mimic the two extreme lipid phases that occur in biological membranes: the gel phase (*L*_β_, also called solid ordered, *s*_o_ phase) and the fluid phase (*L*_α_, also called liquid disordered, *l*_d_ phase).^[^ ^5^, ^6^, ^12^ ^]^ Another essential lipid transient phase occurring in biomembranes, responsible for a variety of membrane‐associated biological activities,^[^ ^10^, ^13^ ^]^ is the liquid ordered (*l*_o_ phase). This phase is exclusively observed at high concentrations of cholesterol (Chol, ≥30 mol%)^[^ ^14^ ^]^ and shares the characteristics of both the gel and fluid phases; it is well ordered, as in the gel phase, but the lipids exhibit a liquid‐like lateral diffusion, resembling the fluid phase. While many studies have focused on the influence of drugs and bioactive compounds in the *l*_d_ phase of the membrane,[^6^, ^15^] limited progress has been made regarding their effects in the more ordered *s*_o_ and *l*_o_ phases. These phases are expected to coexist in different physiological contexts and are particularly important as models of heterogeneity in fluid biological membranes.^[^ ^16^ ^]^

The interaction between drugs or other bioactive compounds and biomimetic membrane models has heavily relied on fluorescence‐based methods, often used to characterize the membrane's biophysical changes induced by these interactions. However, the utilization of fluorescence labeling in this context raises important concerns. The introduction of a fluorescent label can itself induce structural changes in the membrane model system. Also, the intrinsic fluorescence of the drugs or bioactive compounds under investigation can lead to artifacts caused by interference effects like quenching or signal overlap. These factors emphasize the importance of seeking label‐free alternatives to characterize the interaction of drugs or bioactive compounds with biomimetic interfaces.^[^ ^17^ ^]^ Furthermore, to gain a deeper understanding, it is crucial to monitor these interactions both in steady‐state and real‐time modes. Steady‐state analysis offers insights into interactions at an equilibrium state, whereas real‐time monitoring allows tracking changes from nonequilibrium to equilibrium states.^[^ ^18^ ^]^

The present study takes a novel approach to study interactions between three bioactives with varying octanol/water lipophilicities–diclofenac (DCF), caffeine (CAF), and testosterone (TST)–and biomimetic membrane models composed of DPPC or DPPC:Chol(2:1), selected to mimic the *s*_o_ and *l*_o_ lipid transient phases of biomembranes. CAF and TST are hydrophilic and lipophilic model compounds, respectively, recommended by OECD guidelines for in vitro studies for multiple purposes, whereas DCF is a very‐well studied compound and was used as a reference drug for the proposed label‐free approach.

To accomplish this approach, different label‐free characterization techniques were employed, including quartz crystal microbalance with dissipation monitoring (QCM‐D), surface plasmon resonance (SPR), derivative spectroscopy, attenuated total reflection–Fourier transform infrared (ATR–FTIR) spectroscopy, and small‐ and wide‐angle X‐Ray scattering (SWAXS). QCM‐D and SPR are surface‐sensitive techniques that can monitor the real‐time dynamics of supported lipid bilayers (SLBs) assembly and conclude about the interactions resultant from protein or drug adsorption using appropriate models. While QCM‐D simultaneously measures two output signals from a piezoelectric quartz crystal oscillator sensor (i.e., frequency and dissipation), SPR senses changes in the effective refractive index affecting the resonant angle at which a gold crystal can be excited.^[^ ^19^ ^]^ Derivative spectroscopy, ATR–FTIR, and SWAXS are biophysical techniques widely employed to monitor the steady‐state interaction between bioactive compounds and lipid biomimetic systems. While derivative spectroscopy is widely used for determining bioactives partition coefficients in lipid/aqueous media,[^2^, ^6^, ^8^, ^9^] ATR–FTIR and SWAXS are employed for structural characterization of the effects induced by bioactives in the lipid biomimetic systems.[^2^, ^6^, ^20^]

The novelty of the present work lies on multiple levels. 1) it explores the effects of poorly studied bioactive compounds (such as CAF and TST) and a very‐well studied drug (DCF) in the often‐neglected yet highly significant *s*_o_ and *l*_o_ phases of physiological membranes, in contrast to typical studies on drug–membrane interactions. 2) it introduces a cutting‐edge multitechnique approach capable of providing both steady‐state and real‐time information, while effectively circumventing the limitations commonly associated with widely used fluorescent techniques. Furthermore, it explores the effect of clinically relevant concentrations of the bioactives studied. The concentrations of CAF and TST studied (80 and 20 μM, respectively) were chosen to be representative of a dose where toxicological symptoms often begin, while not excessively surpassing the bioactive plasmatic levels.^[^ ^21^ ^]^ The DCF concentration (800 μM) was chosen to be relevant in a context of chronic usage and toxicity level and it is in the range of what can be predicted locally in the intestine after therapeutic oral dosing.^[^ ^22^ ^]^

## 2 Results

### 2.1 Distribution of Bioactive Compounds in the Biomimetic Membrane Models

**Figure 1:**
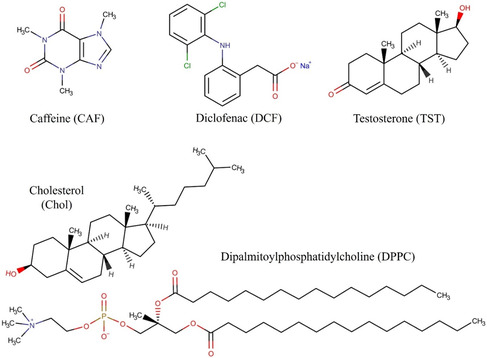
Chemical structures of bioactives and constituents of lipid biomimetic systems used in this study.

The respective chemical structures of both bioactives and lipid biomimetic systems constituents, used in this study, are presented in **Figure** **1**.

The first step in studying the interaction of drugs or other bioactives with biomembranes should be to determine their distribution coefficient (*K*_D_, normally expressed as log*K*_D_) between the lipid and aqueous phases.^[4b]^ In this work, the *K*_D_ of the bioactives in the biomimetic membranes models of DPPC and DPPC:Chol(2:1) were determined using a well‐established derivative spectroscopic method.^[9a]^ **Table** **1** presents the experimental log*K*_D_ values for each bioactive compound determined in vitro in the biomimetic membrane models of DPPC or DPPC:Chol(2:1), alongside the theoretical values determined in silico (MarvinSketch).

**Table 1:** log*K*_D_ values of CAF, DCF, and TST determined in silico and in vitro in biomimetic membrane models of DPPC and DPPC:Chol(2:1)

Bioactive compound	log*K*_D_ experimental values in vitroa)		log*K*_D_ theoretical values in silicob)
	DPPC	DPPC:Chol(2:1)	
CAF	4.13 ± 0.20***^,ns^	4.06 ± 0.19***	−0.55
DCF	3.33 ± 0.11**^,ns^	3.34 ± 0.18**	1.44
TST	3.17 ± 0.20^ns,ns^	3.54 ± 0.08^ ns^	3.37

a) obtained from derivative spectroscopic method in lipid vesicles;

b) calculated using MarvinSketch; statistical significance was determined using a one‐way analysis of variance and Tukey pos*t*‐test for the following comparisons of log*K*_D_ values: experimental versus theoretical (first superscript), values obtained in DPPC versus DPPC:Chol(2:1) (second superscript). ***p ≤ 0.001; ** *p* ≤ 0.01; ns – non significant.

While the in silico prediction was fairly accurate for TST, significant differences between in vitro and in silico log*K*_D_ values were observed for CAF and DCF. According to in silico theoretical log*K*_D_ values, CAF is classified as hydrophilic (log*K*_D_ < 0), DCF as moderately lipophilic (0 < log*K*_D_ < 3), and TST as lipophilic (3 < log*K*_D_ < 5).^[^ ^23^ ^]^ However, when log*K*_D_ values were determined in the lipid/water systems, regardless of their composition, all three compounds present a lipophilic character (3 < log*K*_D_ < 5).^[^ ^23^ ^]^ This inconsistency between experimental and theoretical approaches is not new and has been found in several previous studies,[^6^, ^24^] highlighting the need for a biomimetic membrane model capable of replicating the chemical and biophysical properties of biomembranes.

### 2.2 Real‐Time Characterization of Interactions Between Bioactive Compounds and Biomimetic Membrane Models

**Figure 2:**
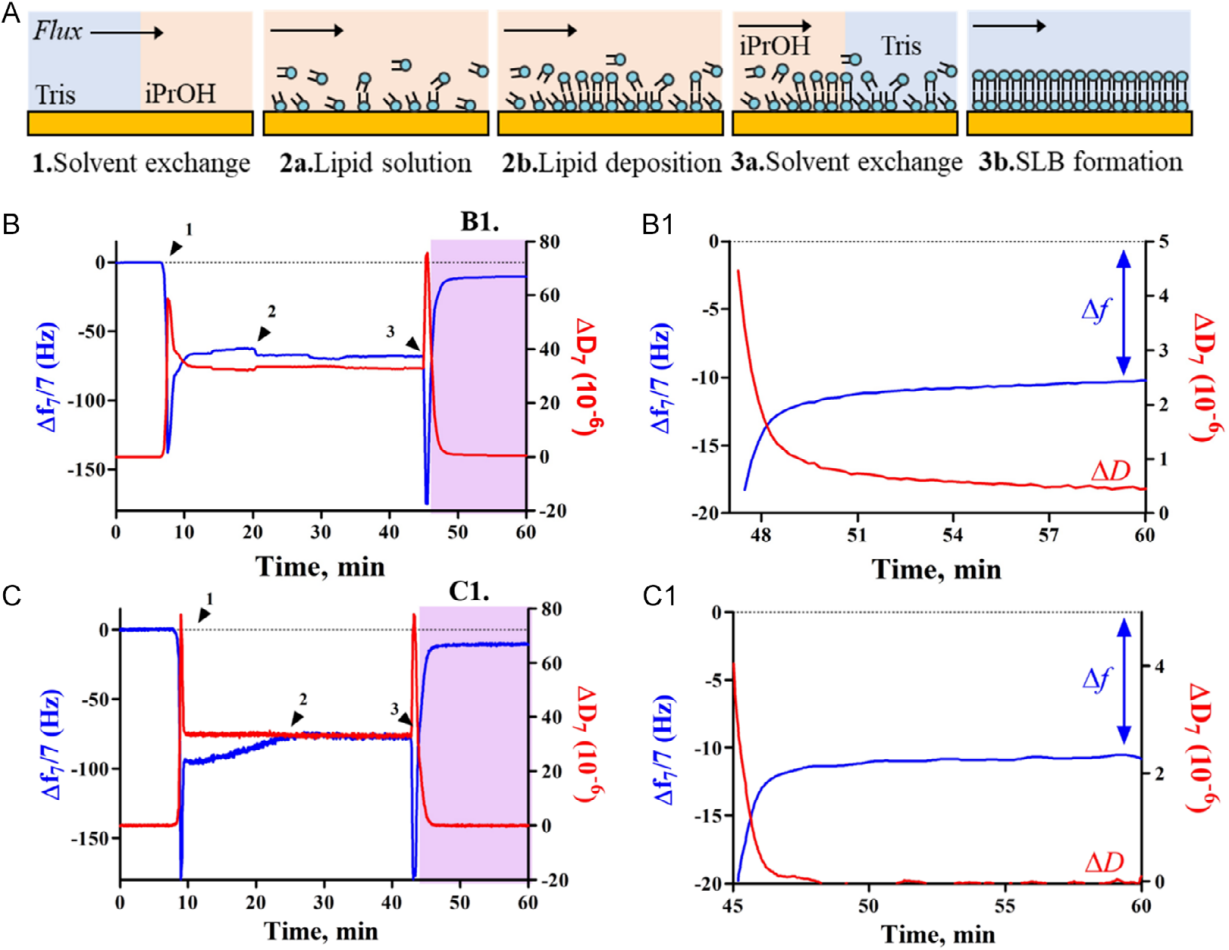
A) Schematic representation of various SALB method stages. B,C) Variations in frequency (*Δf*, blue) and dissipation (*ΔD*, red) for the 7^th^ overtone (35 MHz) during the formation of SLBs of B) DPPC and C) DPPC:Chol(2:1). The arrows indicate key steps in the process: exchange of Tris with isopropanol (arrow 1), injection of a freshly prepared lipid organic solution (arrow 2), and injection of the Tris buffer (arrow 3). The insets (B1 and C1) are scaled differently from the main graphs to provide a clearer visualization of the final *Δf* and *ΔD* values.

QCM‐D is a label‐free technique that monitors 1) changes in oscillation frequency (*Δf*) of a quartz sensor, which indicates mass deposition or removal from this sensor (including water), and 2) changes in energy dissipation (*ΔD*), which are related to the viscoelasticity of the system.^[^ ^25^ ^]^ QCM‐D allowed to follow the formation of SLBs in real time. **Figure** **2** illustrates a typical solvent‐assisted lipid bilayer (SALB) process, and SLB formation on the quartz sensor (Figure 2A), with the corresponding *Δf* and *ΔD* profiles obtained for SLBs of DPPC or DPPC:Chol(2:1) (Figure 2B,C, respectively). The in situ assembly of SLBs using the SALB method can be broken down into several steps: first, a baseline is established using the aqueous solvent (Tris buffer), followed by its replacement with an organic solvent (isopropanol) (Figure 2, step 1). This solvent exchange results in a steep decrease in *Δf* and an increase of *ΔD*, followed by signal stabilization within 20 min. The following injection of the lipid solution in isopropanol (Figure 2A, step 2a) results in lipid deposition on the QCM crystal (Figure 2A, step 2b) with a subsequent new exchange for the aqueous solvent (Figure 2A, step 3a) and formation of SLB (Figure 2A, step 3b).The acoustic responses of QCM‐D were consistent with the expected profiles of lipid deposition on the gold substrate using the SALB method.^[^ ^26^ ^]^ Accordingly, *Δf* averaged at −10 and −12 Hz, with negligible *ΔD* values, and the multiple frequency overtone signals overlap at the end of the SALB method (Figure S1, Supporting Information): all these factors are consistent with the typical QCM‐D fingerprint of fully formed single SLBs[^17^, ^27^] of DPPC or DPPC:Chol(2:1) (Figure 2, insets B1 and C1, respectively and Figure S1, Supporting Information).

These results were complemented with SPR analysis of the characteristic reflectivity changes of incident light (Figure S2, Supporting Information): the peaks of minimum reflectivity for the DPPC bilayer were found to be on average at 69.48° and 66.21°, for laser wavelengths of 670 and 785 nm, respectively. These values represent increments of 0.13° (670 nm) and 0.08° (785 nm) compared to the unmodified gold sensor, which is consistent with the deposition of 112 ng cm^−2^ of lipid over the gold sensor. *n* of 1.423 for DPPC, similar to previously reported data,^[^ ^28^ ^]^ was calculated. In contrast, the calculated *n* for DPPC:Chol(2:1) was lower (1.316 ± 0.058) when compared to DPPC.

**Figure 3:**
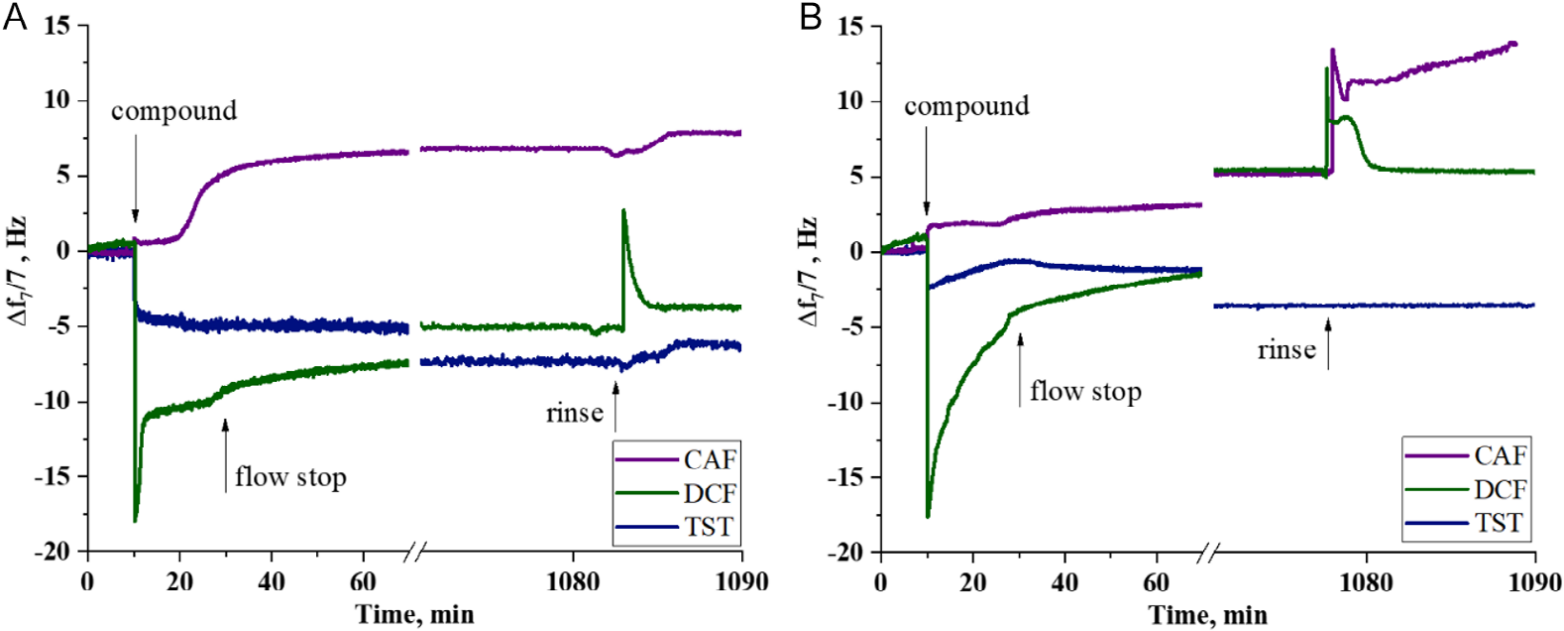
Variations of frequency upon the interaction of CAF (purple), DCF (green), and TST (blue), with A) DPPC and B) DPPC:Chol(2:1) SLBs. The timepoints of compound injection, flow stop, and final rinsing are indicated by the arrows.

After SLB formation, three bioactive compounds, CAF, DCF, and TST, were incubated with the biomimetic membrane models and the *Δf* monitored as function of the time is presented (**Figure** **3** and Figure S3, Supporting Information).

The variations of mass (*Δm*) and *n* induced by these incubations were compared after ≈18 h (**Table** 2). Because the formed SLBs consist of lipid ordered phases (*s*_o_ and *l*_o_ phases for DPPC and DPPC:Chol (2:1), respectively) with some degree of rigidity, *Δm* was estimated from the QCM‐D data using the Sauerbrey model for rigid layers (Equation (1)), which has been previously validated for lipid bilayers on surfaces^[^ ^29^ ^]^

(1) $$\Delta m=\frac{-c\times \Delta f_{n}}{n_{i}}$$

where *Δf*_n_ is the frequency of overtone *n*_i_ and *C* = 17.7 ng·Hz^−1^ cm^−2^.

**Table 2:** Estimated final variation in mass (*Δm*) and refractive index (*Δn*) of DPPC and DPPC:Chol(2:1) SLBs after 18 h incubation with CAF, DCF, and TST.

Compound	DPPC		DPPC:Chol(2:1)	
	Δ*m* (%)a)	Δ*n* (RIU)b)	Δ*m* (%)a)	Δ*n* (RIU)b)
CAF (*n = 1.68*)^c)^	−53	−0.19	−85	−0.06
DCF (*n = 1.66*)^c)^	22	0.09	−14	−0.02
TST (*n = 1.56*)^c)^	41	0.05	35	0.04

a) Experimental values by QCM‐D.

b) Values obtained by SPR experiment.

c) Values obtained from ChemSpider database (Royal Society of Chemistry).

The introduction of CAF (Figure 3A,B) resulted in increased *Δf* for both lipid systems, indicating mass loss. After rinsing, for DPPC, there was a 53% reduction in total mass and a corresponding 0.19 decrease in *n.* Similar results were obtained for the DPPC:Chol(2:1) system, with a mass loss of 85% and a decrease of 0.06 in *n* (Table 2). These results suggest that the interactions between CAF and the lipids are stronger than the hydrophobic interactions holding the bilayers, leading to the removal of lipid molecules from the bilayer. This phenomenon is commonly observed in biological systems, wherein specific interactions with the substrate deposited on the QCM crystal result in material depletion from the crystal surface.^[^ ^30^ ^]^

The interactions between the biomimetic membrane models and DCF were dependent on the bilayer composition (Figure 3A,B, Table 2). In the case of DPPC, the introduction of DCF caused a 22% increase in total mass and an increase of *n* of 0.09, while for DPPC:Chol(2:1) SLBs, there was a 14% mass loss accompanied by a decrease in *n* of 0.02 (Table 2). However, it is noteworthy that the initial interaction of DCF with SLBs caused a fast and substantial decrease in *Δf* (about −18 Hz on both SLBs), followed by a continuous material depletion throughout the experiment, with differing amplitudes for DPPC and DPPC:Chol(2:1) (Figure 3A,B, green data).

The introduction of TST to the DPPC SLB caused an initial rapid decrease in *Δf* (−5 Hz), indicating mass deposition (Figure 3A, blue line), followed by a steady, smaller decrease in *Δf* related with the reorganization of the deposited mass and adsorption of additional TST. At the end of the process and after rinsing, the total deposited mass increased by 41% (Table 2). A similar behavior was observed in the case of DPPC:Chol(2:1) (Figure 3B, blue line), where the total deposited mass increased by 35% at the end of the experiment. These observations are consistent with SPR results: on DPPC, TST increased *n* by 0.05, while on DPPC:Chol(2:1), *n* increased by 0.04 (Table 2).

### 2.3 Steady‐State Characterization of the Interactions Between Bioactive Compounds and Biomimetic Membrane Models

While QCM‐D and SPR provide real‐time information on interactions between bioactive compounds and lipid interfaces, SWAXS and ATR–FTIR can give steady‐state information about bilayer order, hydrocarbon chain packing, and bilayer reorganization, offering a complementary characterization of the effects induced by the bioactive compounds.

**Figure 4:**
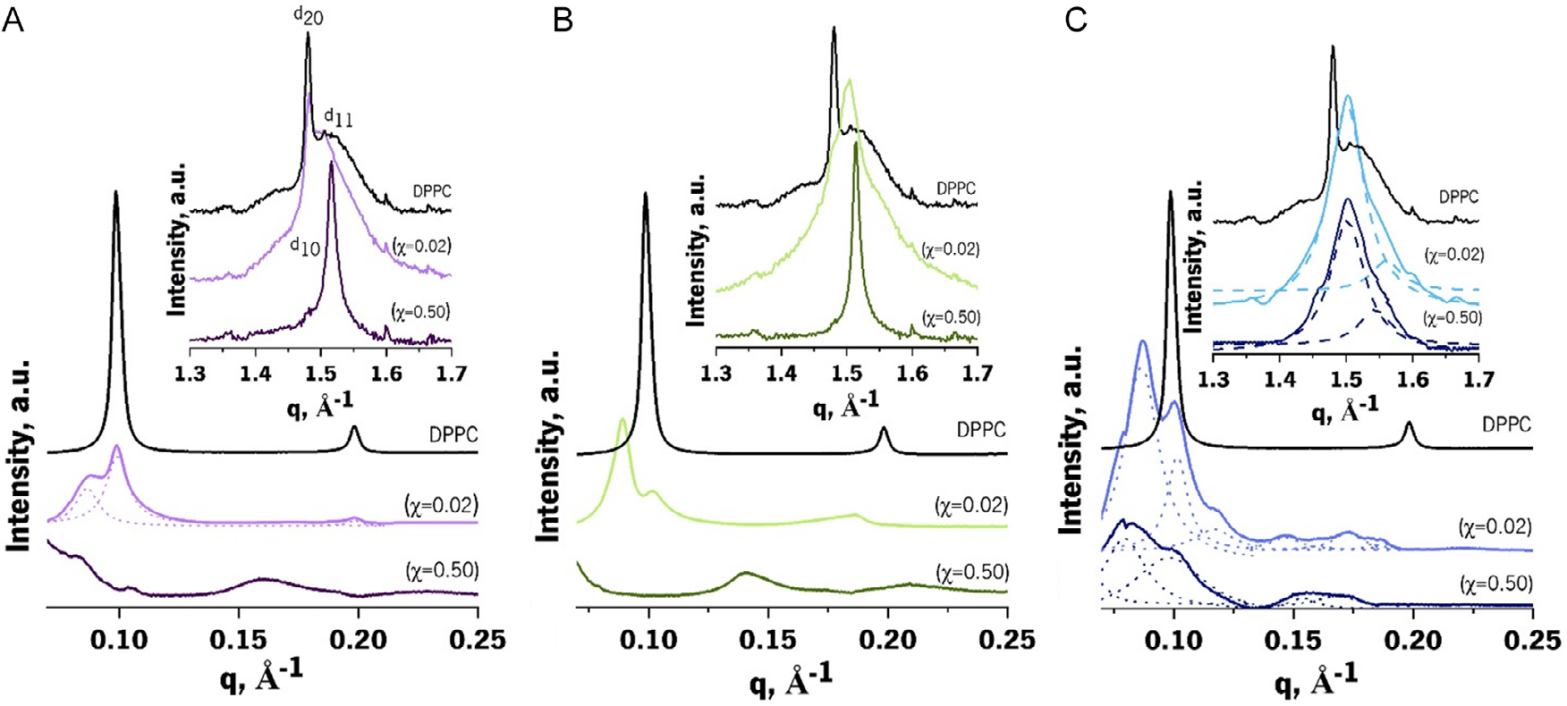
SWAXS diffractograms (SAXS in the main graphs below and WAXS in the insets) of DPPC in the absence (black lines) and in the presence of increasing molar ratios (χ = 0.02 and 0.50) of CAF (A, purple shades), DCF (B, green shades), and TST (C, blue shades).

The pure DPPC system exhibited a common SAXS pattern (**Figure** **4**, black line), with lamellar structure spaced by 63.52 Å (sum of the thickness of DPPC bilayer and the hydration layer) and a full width at one‐half of their intensity (FWHM) of 1329 Å^−1^, which are consistent with the literature.^[6a]^

Increasing amounts of bioactive compounds in the pure DPPC systems, mimetic of the *s*_o_ lipid phase, resulted in significant changes in the SAXS pattern, affecting shape, position, and/or number of Bragg peaks (Figure 4, main graphs). The sharp single Bragg peak observed for DPPC (Figure 4, main graphs in black) changed into a convoluted peak shifted to higher long‐spacing distances when CAF was incorporated into the biomimetic membrane model at a molar ratio of 0.02 (Figure 4A, main purple graph with χ = 0.02). These changes indicate the coexistence of two lamellar phases: a pure DPPC phase at a repeated distance of 63.29 Å and a CAF‐affected phase with an increased repeated distance of 72.55 Å. At a higher CAF molar ratio, the diffractogram presented a broad and low‐intensity Bragg peak, shifted toward longer‐spacing distances (Figure 4A, main purple graph with χ = 0.50). The asymmetrical and diffuse dispersion pattern may be a consequence of deep changes in lipid order and hydration, potentially leading to the loss of lipid organization in multistacked layers or its reassembly into vesicles, as previously observed for other lipid matrices.^[^ ^31^ ^]^ Additionally, the incorporation of CAF at molar ratio of 0.02 also decreased the averaged correlation length values from 1329 to 541 Å^−1^, indicating its impact on the cooperative unit of the lipid bilayers, coherent with its reported shallower membrane location at the polar/nonpolar lipid interface.^[^ ^32^ ^]^

Incorporating DCF into the DPPC system resulted in the coexistence of two lamellar phases (a pure DPPC phase and a DCF‐affected phase), with a shift to higher long‐spacing distances (Figure 4B, main green graph with χ = 0.02). At the highest molar ratio (Figure 4B, main green graph with χ = 0.5), the asymmetric and diffuse scattering patterns indicate that DCF causes substantial changes in lipid order and hydration, resulting in the loss of lipid organization or its reassembly into vesicles, similar to the effects observed with CAF. A reduction of the correlation length value was also observed with increasing DCF content, from 1329 Å^−1^ (pure DPPC) to 388 Å^−1^ for DCF molar ratio of 0.02. These changes reflect alterations at the membrane cooperative unit level, indicating interaction of DCF with C1–C8 of lipid acyl chains, in agreement with its reported location.[^6^, ^15^]

The incorporation of TST into the DPPC system resulted in significant changes in the lattice parameters. At molar ratios of 0.02 and 0.5, a lamellar phase (ratio of Bragg peaks position 1:2) coexists with a hexagonal phase (ratio of Bragg peaks position 1:√3) (Figure 4C, main blue graphs with χ = 0.02 and χ = 0.5). These changes in the lattice parameters are attributed to the insertion of TST into the hydrophobic domains of the bilayer, close to the hydrophobic–hydrophilic region. This induces changes in lipid packing, affecting the order of the lipid acyl chains as well as the interactions between the lipid headgroups. This behaviour is similar to the insertion of Chol into the membrane,^[^ ^33^ ^]^ which has a sterol moiety in common with TST.

WAXS diffraction patterns for pure DPPC showed an asymmetric double‐Bragg peak (assigned as d_20_ and d_11_), characteristic of pseudohexagonal tilted chain packing with short‐spacing distances of 4.24 and 4.14 Å (Figure 4, inset black graph). This corresponds to a cross‐sectional area of 20.11 Å^2^, which is in good agreement with previous reports.^[6a]^ In the presence of the bioactive compounds, the asymmetric double Bragg peak is converted into a single peak (assigned as d_10_), characteristic of nontilted hexagonal chain packing (Figure 4, insets A–C).

**Figure 5:**
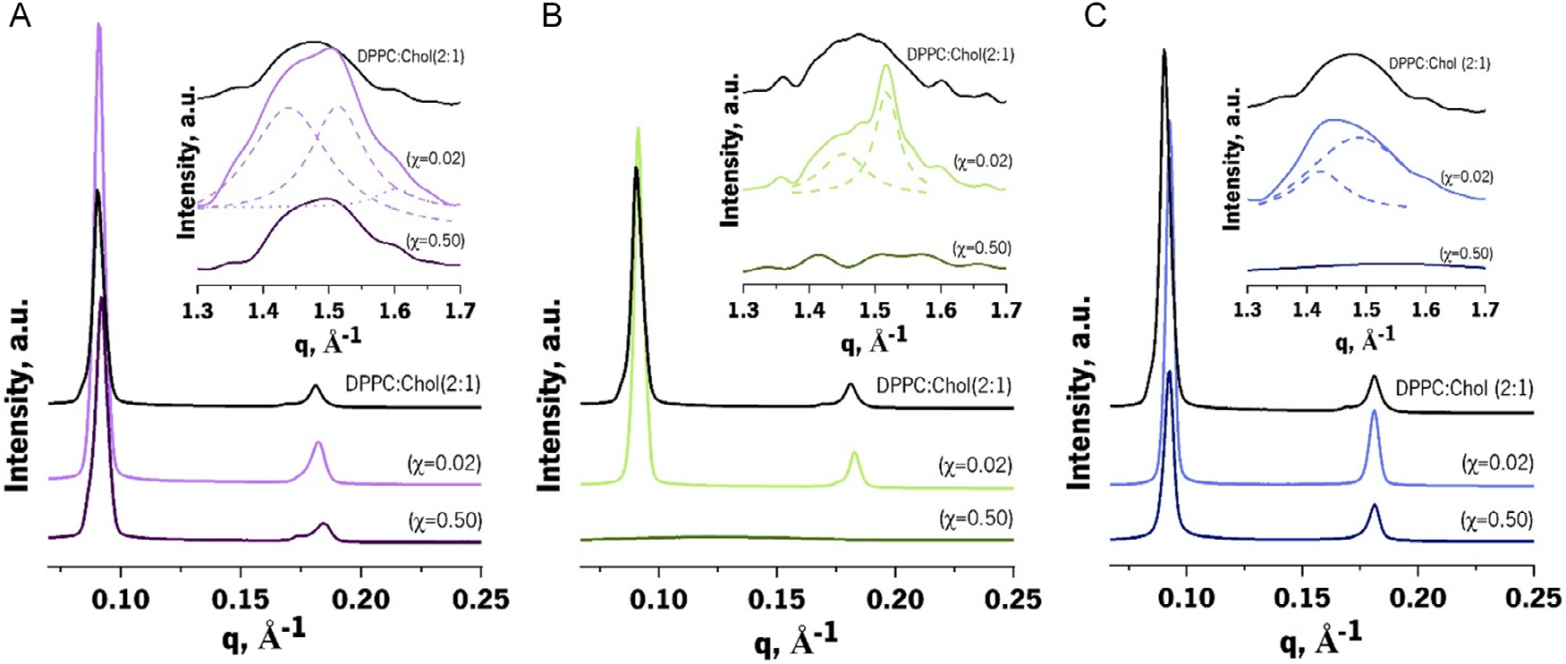
SWAXS diffractograms (SAXS in the main graphs below and WAXS in the insets) of DPPC:Chol(2:1) in the absence (black lines) and in the presence of increasing molar ratios (χ = 0.02 and 0.50) of CAF (A, purple shades), DCF (B, green shades), and TST (C, blue shades).

The SAXS pattern of DPPC:Chol(2:1) exhibited a lamellar structure spaced by 69.35 Å, with a correlation length of 1083 Å^−1^ (**Figure** **5**, main graphs in black). The tilt loss of acyl chains toward a stretched position in *lo* phase that happens during Chol incorporation^[^ ^34^ ^]^ can explain the increase in 6.1 Å found when compared to DPPC.

The SAXS patterns of DPPC:Chol(2:1) were less affected by the addition of the bioactive compounds, especially at the lower bioactive:lipid molar ratios (Figure 5A–C main graphs, χ = 0.02), where only minor reductions in long‐spacing and correlation length were observed. However, at the highest molar ratio, both CAF and DCF induced notable changes in the SAXS diffraction pattern (Figure 5A,B main graph, χ = 0.5). CAF reduced the correlation length and caused some phase separation effects, which were more noticeable in the second‐order diffraction (Figure 5A main graphs, χ = 0.5). DCF had an even more pronounced effect on the diffraction pattern, indicating a strong lipid disordering effect that destroys the multistacked assembly of the bilayers (Figure 5B main graph, χ = 0.5).

The WAXS diffraction pattern of DPPC:Chol(2:1) (Figure 5, inset graphs in black) displayed a single and broad Bragg peak, which is consistent with the effect of Chol in the *s*_o_ phases of DPPC. This Chol‐induced effect resulted in decreased lipid order and cohesion forces, ultimately abolishing the phase transition. The incorporation of the bioactive compounds at a low bioactive:lipid molar ratio (Figure 5A–C inset graphs, χ = 0.02) resulted in convoluted Bragg peaks, indicating a partial recovery of the tilted pseudohexagonal packing. This suggests that all the bioactive compounds interact with the headgroup region of the lipids. At higher molar fractions of the bioactive compounds, the broad WAXS diffraction returned, indicating a reduction in lipid order and cohesion forces and the abolishment of the phase transition. Taken together, these differences observed in SAXS and WAXS patterns demonstrate that all bioactive compounds interacted with the membrane mimetic models of DPPC:Chol(2:1).

**Figure 6:**
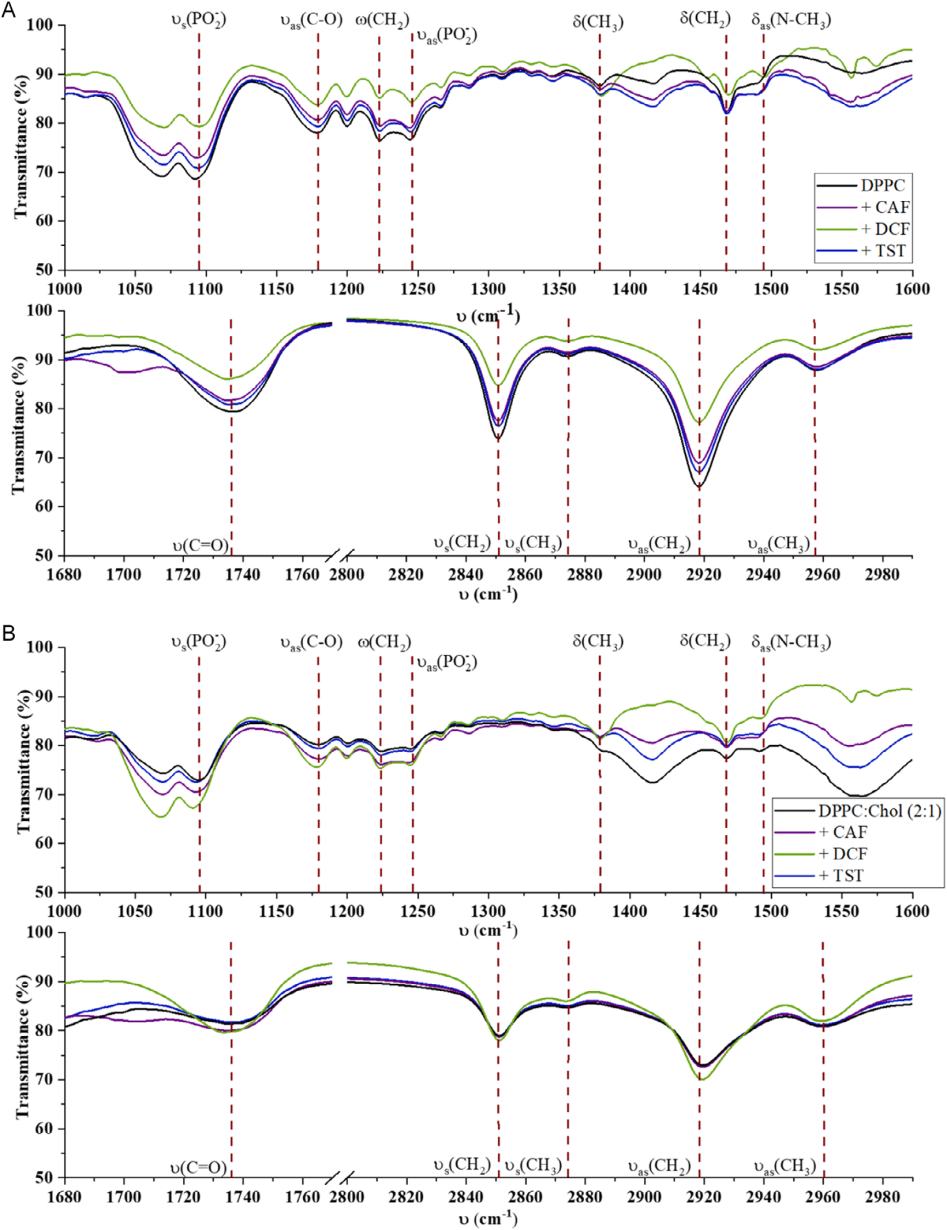
ATR–FTIR spectra in the range of 1000–1600 cm^−1^ and 1690–2990 cm^−1^ for biomimetic membrane models composed of A) single‐lipid DPPC and B) binary mixture DPPC:Chol(2:1) in the absence (black lines) and presence of CAF (purple line), DCF (green line), and TST (blue line). Vibrational modes: as = asymmetric; s = symmetric; *υ* = stretching; *δ* = in plane bending.

ATR–FTIR spectroscopy is a valuable tool for studying interactions at the lipid–water interface, providing information about the chemical groups of phospholipid constituents in biomimetic membrane models, their dynamic behavior, and the effects of drug or other bioactive compound interactions.^[^ ^20^, ^35^ ^]^ **Figure** **6** depicts the ATR–FTIR spectra of DPPC and DPPC:Chol(2:1) in the regions of 1000–1600 cm^−1^ and 1680–2990 cm^−1^. The main peak assignments are listed in Table S1, Supporting Information.

In the acyl chains, which constitute the hydrophobic region of a lipid bilayer, the CH_2_ symmetric (ν_s_(CH_2_)) and asymmetric stretching (ν_as_(CH_2_)) vibrations around 2850 and 2929 cm^−1^, respectively, are typically the most prominent bands in IR lipid spectra.^[^ ^36^ ^]^ The ν_s_(CH_2_) band is highly sensitive to changes in mobility and conformational disorder of hydrocarbon chains.[^15^, ^37^] In Figure 6A (lower graphs), the ν_s_(CH_2_) and ν_as_(CH_2_) absorption bands of the DPPC system are observed at 2851 and 2919 cm^−1^, respectively, consistent with the literature.[^15^, ^37^] The addition of Chol (Figure 6B, lower graph) resulted in slight upward shifts to 2852 and 2921 cm^−1^, for ν_s_(CH_2_) and ν_as_(CH_2_), respectively, suggesting a higher fluidity of the DPPC:Chol(2:1) membrane models. Notably, no changes in these bands were observed upon introduction of the bioactive compounds into the DPPC (Figure 6A, lower graph) or DPPC:Chol(2:1) (Figure 6B, lower graph) membrane models. ν_as_(CH_3_) and ν_s_(CH_3_), respectively, found at 2958 and 2874 cm^−1^ for DPPC, are also sensitive to conformational changes.^[^ ^36^ ^]^ The absence of significant changes in CH_3_ vibrations upon interaction with the bioactive compounds suggests that none of the compounds are deeply inserted in the membrane. (Figure 6A, lower graph). Upon incorporation of Chol, ν_s_(CH_3_) remained at 2874 cm^−1^, and ν_as_(CH_3_) shifted to 2960 cm^−1^, suggesting some level of conformational changes at the hydrocarbon chains. Nevertheless, no changes were observed upon addition of the bioactive compounds to the DPPC:Chol(2:1) system (Figure 6B, lower graph).

In the range of 1245–1090 cm^−1^ (Figure 6A,B, upper graph), two distinct PO_2_^−^ bands are observed within the headgroup region of both biomimetic membrane models (Figure 6A,B, upper graph): ν_as_(PO_2_^−^) at 1244 cm^−1^ and ν_s_(PO_2_^−^) at 1092 or 1093 cm^−1^ for DPPC or DPPC:Chol(2:1), respectively.[^6^, ^15^, ^38^] The addition of the bioactive compounds shifted the ν_s_(PO_2_^−^) band to higher wavenumbers in DPPC (Figure 6A, upper graph) or lower wavenumbers in DPPC:Chol(2:1) (Figure 6B, upper graph). The incorporation of DCF into DPPC shifted the ν_as_(PO_2_^−^) band to higher wavenumbers (Figure 6A, upper graph), whereas CAF addition into DPPC:Chol(2:1) shifted it to lower wavenumbers (Figure 6B, upper graph). These shifts in the vibrational modes may result from changes in the number and orientation of H‐bonded water molecules around the headgroup or in the environment polarity upon the incorporation of the bioactive compounds.

In the hydrophilic/hydrophobic interface region, the ν(C═O) band (Figure 6A,B, lower graph) reflects the hydrogen bonding of water to carbonyl groups of phospholipids.^[36a]^ The deconvolution of the main band assigned to ν(C═O) vibration (Figure S4, Supporting Information) revealed two components: ν(C═O_free_) at higher wavenumbers (1740–1742 cm^−1^) corresponding to the vibrational modes of nonhydrogen bonded conformers and ν(C═O_bond_) at near 1728 cm^−1^ attributed to hydrogen bonded conformers.[^36^, ^37^]

A relationship between band conformers’ position and relative area (proportional to the number of conformers)^[^ ^37^ ^]^ was established, and intensity ratios of the most important bands were compared (**Table** **3**). The *I*[*ν*_as_(CH_3_)]/*I*[*ν*_s_(CH_2_)]) ratio provides a general view of the microviscosity of the hydrocarbon chains, and *I*[*ν*_s_(CH_3_)]/*I*[*ν*_s_(CH_2_)]) ratio reflects the chain decoupling effect. As the chains decouple, (e.g., by intermolecular interactions decrease), the rotational and vibrational freedom of the terminal CH_3_ increases, leading to an increased ratio value. The *I*[*ν*_as_(CH_2_)]/*I*[*ν*_s_(CH_2_)] ratio reflects the lateral packing density of the acyl chains and serves as an indicator of lipid order.[^36^, ^37^, ^39^]

**Table 3:** FTIR parameters to predict the interaction of biomimetic membrane models with the bioactive compounds at hydrophilic and hydrophobic regions of bilayer

Biomimetic membrane modela)	Hydrophilic region				Hydrophobic region		
	ν_s_(PO_2_^−^)	*ν*(C═O)_free_Δ*A*	*ν*(C═O)_bond_Δ*A*	Area r$\frac{\text{ν(C}═{\text{O)}}_{\text{free}}}{\text{ν(C}═{\text{O)}}_{\text{bond}}}$	Int. r$\frac{\nu_{\text{as}}{\text{(CH}}_{3})}{\nu_{s}{\text{(CH}}_{2})}$	Int. ns$\frac{\nu_{s}{\text{(CH}}_{3})}{\nu_{s}{\text{(CH}}_{2})}$	$\frac{\nu_{\text{as}}{\text{(CH}}_{2})}{\nu_{s}{\text{(CH}}_{2})}$
DPPC	1090 cm^−1^	1741 cm^−1^−	1728 cm^−1^−	0.3	0.5	0.5	1.4
+ CAF	1093 cm^−1^	1740 cm^−1^64%	1723 cm^−1^−27%	0.7	0.5	0.6	1.4
+ DCF	1094 cm^−1^	1739 cm^−1^5%	1726 cm^−1^−55%	0.7	0.5	0.9	1.5
+ TST	1093 cm^−1^	1741 cm^−1^7%	1728 cm^−1^−22%	0.4	0.5	0.6	1.4
DPPC:Chol(2:1)	1093 cm^−1^	1741 cm^−1^−31%	1722 cm^−1^−47%	0.4	0.9	1.0	1.3
+ CAF	1092 cm^−1^	1740 cm^−1^28%	1720 cm^−1^35%	0.4	0.9	0.9	1.3
+ DCF	1090 cm^−1^	1741 cm^−1^6%	1728 cm^−1^47%	0.3	0.8	0.8	0.9
+ TST	1092 cm^−1^	1742 cm^−1^−20%	1725 cm^−1^15%	0.3	0.9	0.9	1.3

a) *A* = Area of the band; *I* = Intensity of the band; Δ*A* = difference between the band areas of the membrane models with and without the bioactive compound; Vibrational modes: as = asymmetric; s = symmetric; *ν* = stretching.

According to Table 3, the incorporation of Chol resulted in a substantial decrease of both ν(C═O) conformers, accompanied by a downward shift of the ν(C═O_bond_) vibration. This observation, along with the dehydration observed by the upward increase in *ν*_s_(PO_2_^−^), and the increased A[*ν*(C═O_free_)]/A[*ν*(C═O_bond_)] ratio, suggest the formation of hydrogen bonds between Chol and the polar headgroup of DPPC, in line with the umbrella model of Chol interaction with lipids.^[^ ^40^ ^]^ Furthermore, the increased *I*[*ν*_as_(CH_3_)]/*I*[*ν*_s_(CH_2_)] ratio and decreased *I*[*ν*_as_(CH_2_)]/*I*[*ν*_s_(CH_2_)] ratio suggest increased membrane fluidity and rotational motion and reduced lateral packing (Table 3).

In the case of the DPPC model, CAF increased free conformers (Table 3), likely due to the disruption of hydrogen bonds between carbonyl groups and water molecules, potentially forming new bonds with CAF moieties. The substantial increase in the A[*ν*(C═O_free_)]/A[*ν*(C═O_bond_)]) ratio (Table 3) also suggests that the primary interaction site of CAF with the membrane is at the interfacial region involving carbonyl groups. In terms of indicators in the hydrophobic region (Table 3), CAF has no effect on the overall microviscosity or lateral packing of DPPC bilayers, although it does increase chain decoupling. When interacting with DPPC:Chol(2:1) biomimetic membrane model, CAF also increased ν(C═O_free_) conformers, but less extensively, while simultaneously increasing the bonded conformers. This suggests that although the increased *ν*(C═O_free_) conformers are related to the disruption of hydrogen bonds between carbonyl groups and water, the shift of *ν*_s_(PO_2_^−^) to lower frequencies indicates an increased hydration degree. These findings imply that CAF and Chol compete for binding to C═O at the interfacial region, making this region less accessible to CAF, as evidenced by a lower A[*ν*(C═O_free_)]/A[*ν*(C═O_bond_)]) ratio when CAF is integrated into DPPC:Chol(2:1), compared to when it is incorporated into DPPC alone (Table 3). CAF had no effect on the overall fluidity or lateral packing of DPPC:Chol(2:1) bilayers, but decreased chain decoupling, which is consistent with its promotion of hydration at the hydrophilic to hydrophobic interface while inducing *gauche* defects in the hydrophobic region.^[^ ^32^ ^]^

The addition of DCF to the DPPC system resulted in a downward shift of ν(C═O) conformers, particularly the bonded ones, as well as in an increased A[*ν*(C═O_free_)]/A[*ν*(C═O_bond_)]) ratio and an upward shift in *ν*_s_(PO_2_^−^). (Table 3). These findings suggest that DCF interacts at the hydrophobic/hydrophilic interface, affecting the hydration layer of the membrane. Although DCF had no effect on the overall microviscosity of DPPC hydrocarbon chains, it did increase acyl chain rotational motion and lateral packing (Table 3). In DPPC:Chol(2:1) biomimetic membrane models, DCF decreased the frequency of ν_s_(PO_2_^−^) and shifted ν(C═O_bond_) to higher frequencies, while *ν*(C═O_free_) conformers remained unchanged, confirming its location at both polar headgroup and interfacial regions. However, the decreased A[*ν*(C═O_free_)]/A[*ν*(C═O_bond_)]) ratio suggests that DCF interacts more superficially at the water/headgroup region than at the interfacial region. The increased hydration and interaction at this more superficial region may explain the reduction in lateral packing and the slight reduction in fluidity of DPPC:Chol(2:1) membranes.

Although the interaction of TST with the membrane models of DPPC did not change the frequencies of *ν*(C═O), slight changes in the conformers were observed. A slight increase in the A[*ν*(C═O_free_)]/A[*ν*(C═O_bond_)]) ratio suggests a weak TST interaction at the carbonyl groups level (Table 3). Additionally, the upward shift of *ν*_s_(PO_2_^−^) to 1093 cm^−1^ indicates dehydration at the polar headgroup, implying the formation of new hydrogen bonds between TST and the phosphate group. Therefore, TST likely interacts with polar headgroups in a superficial region but only partially at the polar/nonpolar interface. This is supported by the effects of TST in the hydrophobic region (Table 3). Indeed, TST slightly decreased intermolecular interactions without affecting the microviscosity or lateral packing of DPPC membranes. These findings are also consistent in the hydrophobic region of DPPC:Chol(2:1), where TST does not disturb the general microviscosity or lateral packing of the bilayers (Table 3). Regarding the interaction at the hydrophilic region, Chol appears to enhance interaction of TST closer to the interfacial region. This is supported by increased hydration of the phosphate group, indicated by a slight downward shift of *ν*_s_(PO_2_^−^) and an upward of shift of ν(C═O) conformers, while TST slightly decreases their A[*ν*(C═O_free_)]/A[*ν*(C═O_bond_)]) ratio at the expense of free conformers.

## 3 Discussion

In the present work, we employed label‐free biophysical techniques to investigate the interactions between bioactive compounds and the more ordered *s*_o_ and *l*_o_ phases of biomimetic membrane models, either in real time (QCM‐D and SPR) or in a steady state (derivative spectroscopy, SWAXS, and ATR–FTIR).

Surface analysis techniques like QCM‐D and SPR have been instrumental to follow the in situ formation of model membranes. Reimhult et al.^[^ ^19^, ^41^ ^]^ developed theoretical foundations for studying the time‐dependent behavior of simple lipid bilayers composed of POPC. Parkkila et al.^[17b]^ combined QCM‐D and dual‐wavelength SPR to provide a comprehensive characterization of the formation process of lipid bilayers with various lipid compositions. Here, we demonstrated that the combination of QCM‐D and SPR is not limited to monitoring the formation of membranes but can also be powerful approach to further investigate the interactions between biochemical compounds of interest and in situ assembled SLBs. Furthermore, the continuous flow conditions offered by QCM‐D and SPR are superior in mimicking physiological settings, providing useful insights into time‐correlated physicochemical interactions at the interface between membranes and bioactive compounds/drugs, which are essential for understanding the biological response. The importance of these techniques is evidenced further by the fact that physiological interactions at the membrane level are not static, but dynamic. Indeed, various reports have highlighted that looking at the steady state of a process provides only a snapshot, whereas real‐time approaches give a better understanding of the dynamic interactions under flow conditions.^[^ ^42^ ^]^ In this context, QCM‐D and SPR are recognized as successful approaches. For example, traditional procedures for investigating bacterial, parasite, or cell adhesion are typically performed under static settings and rely on human or semiautomated read‐outs at specific time points that are difficult to standardize. In contrast, QCM‐D and SPR allow for follow‐up of the whole process under dynamic settings (flow) and are sensitive to changes in mass at the order of ng cm^−2^. Because of these advantages, they are increasingly used in biomedical research, allowing for real‐time observation of the biofilm forming process.[^42^, ^43^] Another example where QCM‐D and SPR could be important is in the design of drugs and bioactives, whose therapeutical effect depends on the collective nanoscale physicochemical interactions at the membrane interface. Knock‐in or knock‐out gene expression is frequently used to validate the selectivity of a drug or bioactive for receptor recognition. While such traditional approaches provide information on important molecules for targeting a specific receptor, they do not increase our understanding of the interaction between drugs/bioactives and lipid membranes, which are the barriers more frequently encountered by drugs during body distribution.^[^ ^44^ ^]^ Moreover, by immobilizing on QCM surface membrane models, it can detect the interactions between membranes and bioactives throughout time and under flow conditions, allowing to obtain information about the binding kinetics. Thus, applications in the medical field such as understanding the formation of biofilms and the design of new drugs and bioactives can benefit from these continuous flow approaches.

A comparison between the DPPC model representing the *s*_o_ phase and the binary mixture DPPC:Chol(2:1), which mimics the *l*_o_ domains present in biological membranes, revealed that the effect of Chol depends on the surrounding lipid phase. At temperatures exceeding the lipid's main phase transition temperature (*T*_m_), pure DPPC bilayer is in the liquid disordered phase (*L*_*α*_), characterized by high acyl chain conformation entropy,^[^ ^45^ ^]^ so the phospholipid molecules are loosely packed. Upon introducing Chol into such *L*_α_ phases, hydrogen bonds form between the hydroxyl group of Chol and the phosphate oxygens of the lipid's polar heads, thereby increasing the overall activation energy of the membrane.^[^ ^46^ ^]^ Membrane fluidity is inversely proportional to the activation energy, which implies that the incorporation of Chol into the *L*_α_ phase reduces fluidity. However, when DPPC bilayers are formed and maintained at a temperature lower than the *T*_m_ (e.g., room temperature ≈25 °C; *T*_m_ of DPPC = 41– 42 °C),^[^ ^47^ ^]^ they are in the *L*_*β*_ or *s*_o_ phase, in which the hydrocarbon lipid chains form a densely packed network. Consequently, the lateral lipid diffusion is significantly reduced, and in the *s*_o_ phase, incorporation of Chol has a fluidizing effect by decreasing the van der Waals forces between DPPC molecules.^[^ ^48^ ^]^ Several results are aligned with this phenomenon: SPR showed that SLBs with Chol had lower *n* than pristine DPPC systems, which is consistent with a more liquid‐like behavior. The fact that the DPPC:Chol(2:1) layer has a *n* value lower than 1.33—and most importantly lower than the DPPC layer *n* of 1.423—indicates that the addition of Chol increases the spacing between DPPC lipids, creating empty spaces in the layer, thereby leading to an effectively lowered density. ATR–FTIR confirms the liquid‐like effect of Chol, as DPPC:Chol(2:1) revealed greater chain decoupling and rotational motion of hydrocarbon chains, indicative of increased localized fluidity at deeper areas of acyl chains. Furthermore, WAXS patterns of DPPC:Chol(2:1) bilayers (Figure 5) displayed a broad and diffuse Bragg peak coherent with a decrease in the internal mesophase order and cohesion forces, to the extent that phase transition becomes undetectable. Earlier studies on DPPC bilayers with high Chol content also reported a similar phenomenon.^[^ ^33^ ^]^

CAF is one of the most widely used and safe psychoactive drugs globally, known for promoting wakefulness and attentiveness.^[^ ^32^ ^]^ Although the methods used to evaluate lipophilicity classify CAF as a hydrophilic compound, computational simulations have already reported its ability to distribute through lipid membranes:^[^ ^32^ ^]^ this is in agreement with our real‐time and steady‐state interaction studies, including the derivative spectroscopy results (Table 1), that revealed the lipophilic character of CAF independent of the composition of lipid/water system. When in contact with SLBs, CAF disrupts both single‐lipid and binary‐lipid membrane models, as evidenced by QCM‐D and SPR (Figure 3A, purple curve). In multistacked lipid bilayers, CAF promoted a lipid structural reorganization as observed by SWAXS and FTIR. These findings suggest that CAF localizes just below the DPPC headgroup region at the interfacial region, significantly affecting membrane hydration. This hypothesis for CAF localization aligns well with existing literature, which describes the ability of CAF to attract water to its vicinity, creating water pockets at the hydrophilic/hydrophobic interface and causing overall membrane dehydration and thickening.[^3^, ^27^, ^32^] The reported effects of increased water density at the hydrophilic/hydrophobic region can explain the observed lipid reorganization through curvature changes into vesicular structures, as suggested by SWAXS (Figure 4, main and inset purple graph with χ = 0.5). According to ATR–FTIR studies, CAF induces *gauche* defects in hydrocarbon chains without affecting the overall microviscosity of the biomimetic membrane models. This suggests an increased rotational motion of acyl chains in the proximity of CAF, that is, resulting in a local fluidization effect.[^3^, ^27^, ^32^] However, there are regions of the lipid membrane that remain unaffected, in which the lipid ordered phases are maintained, in agreement with the coexistence of two lamellar spacings observed in SAXS, corresponding to influenced and noninfluenced phases (Figure 4, main purple graph with χ = 0.02).

The local fluidizing effects and the CAF‐induced water pockets reported in literature[^3^, ^27^, ^32^] can also explain why CAF disrupts SLBs: the constant flow experienced in the QCM‐D and SPR techniques, which is absent in the other techniques, may displace lipids due to reorganization and curvature changes induced by CAF. The disruptive effect of CAF in lipid systems is less evident in SWAXS (except for higher molar fractions) than in QCM‐D and SPR (Figure 3A, purple curve). This can be explained through the evident difference between real‐time QCM‐D and SPR and SWAXS steady‐state techniques. While real time follows each event resultant from the interaction of CAF with the lipid system, SWAXS observes it at a final steady‐state point in a closed system, which does not allow the removal of excess bioactive or loosen lipids. Additionally, in multistacked bilayers, lipid systems are more tightly packed than in its supported bilayer form, rendering the interfacial region less accessible to CAF. Nonetheless, the disruptive effect of CAF in multistacked lipid bilayers is visible in SWAXS at higher molar fractions (Figure 4, main and inset purple graph with χ = 0.5).

Interestingly, the disruptive effect of CAF was more pronounced in SLBs of DPPC:Chol(2:1), as evidenced by the increased mass depletion measured by QCM‐D (Table 2). The increased hydration degree caused in DPPC:Chol(2:1) can constitute the main phenomenon beyond the greater disruption effect of CAF in this membrane observed in the real‐time studies. Since that, increased amounts of CAF may lead to an increase in water density at the hydrophilic region, as reported ,[^3^, ^27^, ^32^] it can thus result in curvature changes to vesicular structures that would be dragged off during the final rinsing step. This effect is not noticed in SWAXS and FTIR most likely because the water availability is very limited in these steady‐state setups when compared to the flow‐driven real‐time monitoring techniques. Furthermore, the presence of Chol restricts the access of CAF to the interfacial region, as indicated by FTIR studies. This, combined with the more ordered multistacked bilayers observed in steady‐state techniques, explains why the disruptive effect is only evident in QCM‐D and SPR. The capacity of Chol to promote lipid membrane stability against disruption or limit compounds’ accessibility and/or distribution through lipid membranes has been reported previously.^[^ ^34^, ^49^ ^]^ Nonetheless, both steady‐state techniques indicate that CAF is located near the phosphate headgroup levels, implying that the membrane disruption effect observed by QCM‐D and SPR may be driven by hydration and curvature changes that overcome the Chol protective effect in the less stable supported single‐lipid bilayers.

DCF is one of the most used nonsteroidal anti‐inflammatory drugs worldwide. In contrast to CAF and TST, which are both neutral in aqueous solution, DCF is ionized. The derivative spectroscopy studies indicate that even in an ionized form, DCF is lipophilic and able to interact with the biomimetic membrane models (Table 1). In the presence of DCF, the multistacked lipid bilayers lose their SAXS diffraction, indicating a disruptive membrane effect and a probable lipid assembly in vesicles, which is supported by the preserved WAXS headgroup order (Figure 4, main and inset green graph with χ = 0.5). Although the initial interaction of DCF with DPPC results in a gain of mass due to a quick kinetic‐driven adsorption process, most of this mass is lost over the following hours (Figure 3A, green curve). While this mass cannot be discriminated between DCF or lipid molecules, the SWAXS findings are consistent with membrane disruption and lipid reassembly in vesicular structures and can justify the mass loss due to vesicles and/or bioactive compound being dragged under flow from the crystal's surface. This ability of anionic DCF to destabilize the lipid bilayers was already reported and has been associated with the electrostatic repulsions caused by the high density of negative charges.^[^ ^50^ ^]^

Both SWAXS and FTIR analyses point to DCF location at the hydrophilic/hydrophobic interface, most likely with the negatively charged carboxyl group anchored at the polar headgroup region, in agreement with its reported location.[^6^, ^15^, ^51^] FTIR studies revealed that DCF does not significantly impact the overall microviscosity of DPPC membranes, but at hydrocarbon chains level, this bioactive compound reduces intermolecular interactions, increases rotational motion, and induces a more fluid lateral packing. Therefore, as reported for human erythrocytes,^[^ ^51^ ^]^ the maintenance of overall membrane microviscosity results from a balance between a moderate rigidifying impact of DCF in the polar headgroup region and a moderate disorder effect on the acyl chains.

The membrane disruptive effect of DCF is also observed in the binary‐lipid systems, as evidenced by SWAXS (Figure 5B main graph, χ = 0.5), where the loss of diffraction patterns indicates disruption of the multistacked bilayers, and by QCM‐D and SPR, where the membrane disruption effect is followed by a mass loss (Figure 3B, green curve and Table 2). By interacting with the headgroup region, DCF increases the density of negative charges, disturbing the intermolecular interactions between adjacent lipids and Chol. This possibly breaks the hydrogen bonds formed between the hydroxyl group of Chol and the phosphate oxygens of lipids, ultimately leading to a disruptive effect in the membrane. This charge‐induced effect of DCF on the interaction of Chol with the phospholipid headgroups distinguishes it from CAF. While CAF can disrupt single bilayers (Figure 3B, purple curve and Table 2), the presence of Chol in the more ordered multistacked bilayers renders the interfacial region less accessible to CAF. As a result, only subtle indications of lipid order effects were found for the higher CAF molar fractions in SWAXS (Figure 5A, main graph, χ = 0.5).

TST, a male sex hormone and a cholesterol‐derived steroid,^[^ ^52^ ^]^ is a relatively understudied molecule in terms of its interaction with biomimetic membrane models, with most studies focusing on molecular dynamics simulations.^[^ ^53^ ^]^ Here, our results demonstrate that TST, as a lipophilic molecule, is capable of interacting and become integrated into lipid membranes. This interaction was confirmed by QCM‐D and SPR, reflected by increases in mass and refractive index (*n*), independent of the lipid membrane model (Figure 3, blue curve, and Table 2). SWAXS and FTIR indicated that TST is located at the hydrophobic/hydrophilic interface of the membrane, with greater presence at the headgroup level, which is a typical behavior in steroidal hormone systems.^[^ ^52^, ^53^ ^]^ The presence of TST, characterized by its hydroxyl group protruding into the aqueous phase, induces an expansion of the phospholipid interfacial area. This results in an energetically unfavorable contact between the aqueous and the hydrophobic regions of the acyl chains, leading to the formation of an inverted hexagonal phase, consistent with SAXS measurements (Figure 4C, main blue graphs with χ = 0.02 and χ = 0.5). On the other hand, the presence of TST did not induce changes in the lamellar structure of DPPC:Chol(2:1) system. The intact lamellar structure, with unaltered acyl chain order, supports the notion that Chol plays a stabilizing role in lipid membrane systems reducing the distribution of TST and its influence on membrane permeability. This stabilizing effect was also observed by the lower increase of mass during real‐time measurements, indicating a lower adsorption of TST to the lipid bilayers containing Chol. Similar phenomena have been reported in the literature for interactions between artificial membranes containing Chol and various substances, including ibuprofen,^[^ ^54^ ^]^ ionic liquids,^[^ ^34^ ^]^ antimicrobial peptides,^[49a]^ and several natural compounds.^[^ ^55^ ^]^

Aside from providing information on bioactive–membrane interactions at the molecular level, the combination of real‐time and steady‐state techniques that use SLBs or multistacked bilayers as biomimetic membrane models, respectively, poses some challenges in the interpretation of results that are not fully comparable but complementary. The membrane disruptive effects of the bioactives are more clearly assessed in real time on SLBs (Figure S5A, Supporting Information) than in steady‐state on multistacked bilayers (Figure S5B, Supporting Information). Two reasons can be pointed for this different behavior: 1) multistacked bilayers are more stable to changes than SLBs due to the interbilayer attractive van der Waals interactions and^[^ ^56^ ^]^ 2) flow‐driven real‐time monitoring techniques allow removal of lipids and/or bioactive molecules after they have disrupted the assembled bilayer. Therefore, the disruptive effect of CAF and DCF upon interaction with DPPC:Chol(2:1) is visible in real‐time SLBs (Figure S5A, Supporting Information), but much less so in steady‐state analysis of their interactions with multistacked bilayers (Figure S5B, Supporting Information). Nonetheless, repulsive forces at the headgroup regions can counterbalance the interbilayer attractive van der Waals interactions that confer higher stability to multistacked bilayers.^[^ ^56^ ^]^ As a result, at higher molar ratios, DCF increases the density of negative charges at the headgroup region, interfering with lipid and Chol interactions and causing a visible disruptive effect in multi‐stacked bilayers. If SLBs and flow‐driven real‐time monitoring techniques show more clearly the bioactive‐induced membrane disruptive effects, the steady‐state techniques used herein complement the bioactive‐membrane studies by providing information, like, for example, the membrane location of the bioactives. Accordingly, CAF and DCF have shallower membrane locations near the phosphate headgroup levels, whereas TST is inserted into the hydrophobic domains of the bilayer, close to the hydrophobic/hydrophilic region.

## 4 Conclusion

We have unraveled the intricate molecular interactions between three model compounds and interfaces that mimic biological membranes. The strong coherence between experimental findings from the multitechnique characterization of biomimetic membrane models elucidated compound/membrane interactions with high molecular precision. While no single technique was capable of providing a comprehensive understanding on its own, each approach demonstrated distinct strengths and limitations. QCM‐D and SPR emerged as valuable label‐free techniques for identifying interactions between molecules of interest and a surface. The utility of QCM‐D and SPR in addressing medical concerns is undeniable, and the growing interest in these continuous flow techniques stems from its capacity to test binding and interactions under dynamic conditions in real time. However, although real‐time monitoring of flow‐driven processes is physiologically more relevant, these techniques show only global variations in mass and optical properties and do not discriminate between different types of interactions or relate them to structural reorganization. Therefore, toward the study of such interactions with medical improved relevance, in which human physiological fluid flow is considered, new QCM‐D and SPR protocols would be very useful. On the other hand, ATR–FTIR and SWAXS delivered the most comprehensive information regarding the structural aspects of the biomimetic membrane models before and after interaction, providing a deeper molecular perspective. Nevertheless, these techniques focus on steady‐state effects and do not capture the dynamic processes associated with the distribution of compounds within lipid bilayers, which makes it challenging for the detection of adsorption/desorption phenomena. The findings presented in this study not only contribute to a better understanding of the biophysical aspects of the mode of action of less investigated compounds, such as TST and CAF, but also highlight the significance of using a complementary approach in evaluating membrane biophysical properties and understanding drug–membrane interactions. The role of Chol creating empty spaces in the *s*_o_ bilayer was observed by the reduction of *n* by SPR and is consistent with a more liquid‐like behavior confirmed by ATR–FTIR and WAXS. The use of complementary techniques made possible to uncover intricate details about the molecular processes through which various compounds interact with biomembranes, implying that CAF and DCF had more disruptive membrane effects in shallower locations, whereas TST, inserted deeper into the bilayer's hydrophobic domains, caused less membrane disruption.

We recognize that analyzing the effects of the membrane on the position, orientation, and structure of the drug would be an interesting perspective in terms of potential loss of therapeutic efficacy, and bioaccumulation/toxicity due to changes in the physicochemical properties of drugs triggered by interactions with membranes. Thus, we envisage that the comprehensive insights into specific surface/molecule interactions provided by these multitechnique approaches and other alternative techniques can lead to the development of advanced biodetection and biosensing devices, ultimately enhancing detection capabilities in applications such as in diagnostics and lab‐on‐a‐chip technologies.

## 5 Experimental Section

5.1

5.1.1

##### Materials

DPPC was obtained from Avanti Polar Lipids, Inc. (Instruchemie, Delfzijl, The Netherlands). Chol, DCF, TST, CAF, Trizma base, and isopropanol were acquired from Merck Life Sciences (Algés, Portugal). Gold‐coated AT‐cut quartz sensors (ref. AWS SNS 000043A) were purchased from AWSensors (Valencia, Spain). SPR sensors coated with a layer of gold (≈50 nm) and a chromium adhesion layer (≈2 nm) (ref SPR102‐AU) were acquired from BioNavis (Tampere, Finland). Tris buffer was prepared by dissolving Trizma base (10 mM) in ultrapure water and adjusting the pH to 7.5. All other aqueous solutions were prepared with ultrapure water (Millipore Milli‐Q, resistivity > 18.2 MΩ cm^−1^ at 25 °C).

##### Derivative Spectroscopy

The membrane/aqueous *K*_D_ of each bioactive (DCF, TSF or CAF) was determined using large unilamellar vesicles (LUVs) prepared by the lipid film hydration and extrusion method, as described elsewhere.^[^ ^57^ ^]^ Briefly, DPPC or DPPC:Chol(2:1) was dissolved in chloroform (0.5 mg mL^−1^) and dried under a stream of nitrogen in a rotary evaporator (Buchi R‐200, Sigma‐Aldrich Corp., Buchs, Switzerland). The dried lipid film was hydrated with ultrapure water, at 50.0 °C (a temperature above the main phase transition temperature, *T*_m_, of DPPC)^[^ ^47^ ^]^ and controlled by a thermostatic water bath with stability ±0.1 °C (Unistat CC, Huber, Offenburg, Germany). The lipid colloidal suspensions were then submitted to five alternate cycles of agitation (vortex) and thermostatic bath (50.0 ± 0.1 °C). Finally, the colloidal suspensions were extruded (Lipex extruder Tranferra Nanosciences, Burnaby, Canada) through Whatman Nucleopore polycarbonate filters with pores of 400, 200, and 100 nm (Enzymatic, Loures, Portugal) to obtain LUVs.

Increasing concentrations of LUVs of DPPC or DPPC:Chol(2:1) from 0 to 3,000 μM were prepared in the absence and presence of a fixed concentration of each bioactive compound: DCF (800 μM), TST (20 μM), or CAF (80 μM). The samples were incubated for 30 min and then, the absorption spectra were recorded in the range of 230–550 nm (UV‐3600i Plus spectrophotometer, Shimadzu Corp., Kyoto, Japan) at room temperature (≈25 °C).

The derivative UV–vis absorbance spectra of biomimetic membranes in the absence and presence of each bioactive were analyzed as described elsewhere.[^2^, ^6^] A graphical representation of the total absorbance *D*_T_ as a function of the lipid concentration ([*L*] in mol L^−1^) was obtained. Then, through the application of a nonlinear regression, *K*_D_ was determined from Equation (2).^[8b]^

(2) $$D_{T}=D_{W}+\frac{\left(D_{m}-D_{W}\right)\cdot K_{D}\cdot \left[L\right]\cdot V_{m}}{1+K_{D}\cdot \left[L\right]\cdot V_{m}}$$

where *D*_m_ and *D*_w_ are the absorbances of the bioactive compound in lipid media and aqueous media, respectively, and *V*_m_ is the lipid molar volume (mol L^−1^) calculated from the specific lipid volumes for each artificial membrane system.^[6a]^

Additionally, for comparison purposes, the theoretical values of octanol/water distribution coefficients were calculated using the software MarvinSketch (ChemAxon, Budapest, Hungary).

##### Quartz‐Crystal Microbalance with Dissipation (QCM‐D)

SLBs were prepared in situ by SALB^[^ ^26^, ^58^ ^]^ using gold‐coated quartz crystals as substrate. Before use, crystals were cleaned with acetone, ethanol, and isopropanol for 5 min each in an ultrasound bath at 40 °C. Clean crystals were mounted in separate flow chambers of a Q‐Sense E4 apparatus (Biolin Scientific, Gothenburg, Sweden). Following the SALB method, a baseline was established with Tris buffer, followed by an exchange of the buffer with isopropanol (20 min). Next, fresh solution of DPPC or DPPC:Chol(2:1) in isopropanol (0.5 mg·mL^−1^) was injected for 20 min. Finally, the lipid solution was exchanged with Tris buffer (20 min) to induce the formation of a SLB. After forming the SLB (a stable signal read‐out overlapping for different frequencies and no dissipation), aqueous solutions of DCF (800 μM), TST (20 μM), or CAF (80 μM) were injected for 20 min and then the flow was stopped to study the interactions of the bioactives with the biomimetic membrane models for 18 h. The flow was restarted for a final rinsing step with Tris buffer for 20 min. The variations of frequency (Δ*f*) and dissipation (Δ*D*) observed upon SLB formation and upon interaction of the bioactives were measured in real time at a fundamental resonance frequency of 5 MHz and its overtones (15, 25, 35, 45, 55, and 65 MHz). Controls were made by simultaneously monitoring the adsorption of DCF, TSF or CAF directly on gold. Δ*f* and Δ*D* were averaged with three independent assays. The areal mass (Δ*m*) was calculated using the DFind software (v. 1.2.1, Biolin Scientific, Gothenburg, Sweden).

##### Multiparametric Surface Plasmon Resonance (MP‐SPR)

MP‐SPR (model SPR Navi200, BioNavis, Finland) was used to register changes in the optical properties of DPPC and DPPC:Chol(2:1) SLBs upon interaction with each bioactive (DCF, TSF, or CAF). The flow rate, times, and solutions were replicated from the QCM‐D experiments. Full‐angle scans (40 − 78°) were performed at 670 and 785 nm simultaneously and sensograms were obtained every 60 s. The refractive index (*n*) was calculated following a two‐wavelength approach for the unambiguous estimation of thickness using the LayerSolver software in simulation mode 3 (v. 1.3.8, BioNavis) based on the Fresnel equations, as described elsewhere,^[^ ^59^ ^]^ and setting a water boundary layer of infinite thickness and *n* = 1.33. All conditions were tested in triplicate.

##### Synchrotron Small‐ and Wide‐Angle X‐Ray Scattering (SAXS/WAXS)

For X‐Ray scattering experiments, DPPC or DPPC:Chol(2:1) in the absence or presence of the bioactive compounds (DCF, TST, or CAF) was solubilized in a mixture of chloroform/methanol (9:1 v v^−1^) to obtain different bioactive:lipid molar fractions (*χ* = 0.02 or *χ* = 0.5). The final lipid concentration in each sample was ≈20% w/v. After total dissolution of the compounds, the lipid suspensions were prepared as previously described^[8b]^ and transferred into X‐Ray transparent glass capillaries with 1.5 mm diameter (Hilgenberg, Malsfeld, Germany), which were sealed and stored at 4 °C until measurement. SWAXS measurements were performed at the Austrian SAXS/WAXS beamline at the synchrotron light source Elettra (Trieste, Italy), employing monochromatic synchrotron radiation with wavelength of 1.54 Å and X‐Ray energy of 8 keV. WAXS and SAXS patterns were recorded with a pixel size of 172 μm at positions that covered the typical diffraction spacing (*s*) range (*s* = 2·sin*ϴ*/*λ*, where *λ* is the wavelength and 2ϴ is the scattering angle) using a 2D Pilatus 100 K and 2D Pilatus3 1M detector system, respectively. The lamellar peaks of silver behenate and *p*‐bromo benzoic acid were used as calibrants to determine the diffraction spacings of SAXS and WAXS, respectively. Static exposures were taken at 25 °C controlled by a thermostatic water bath (stability ± 0.1 °C; Unistat CC, Huber, Offenburg, Germany). The data were analyzed as reported previously.[^2^, ^6^]

##### ATR–FTIR

The interactions of the bioactive compounds and biomimetic membrane models were monitored through ATR–FTIR to characterize the vibrational frequencies of chemical groups representative of the main phospholipid regions—hydrophilic head, hydrophobic acyl chain, and the head/tail interfacial region. LUVs with and without CAF, DCF, or TST were prepared as described before, added to crucibles made of aluminum foil, and the aqueous solvent was left to evaporate at 40 °C overnight (Raypa, Barcelona, Spain). ATR–FTIR spectra were acquired with a Spectrum Two Spectrometer (Perkin‐Elmer, Waltham, MA, USA) with a deuterated triglycine sulfate detector and KBr splitter, coupled with an universal attenuated reflectance (UATR) accessory (single‐reflection crystal diamond, Perkin‐Elmer, Waltham, MA, USA). Spectra were acquired at room temperature in the range of 4000–400 cm^−1^, with a resolution of 4 cm^−1^, accumulating 64 scans.

##### Statistical Analysis

Results were reported as a mean ± standard deviation from a minimum of three independent experiments. Statistical analysis was performed using GraphPad Prism Software (v. 6.01 for Windows; GraphPad Software Inc, San Diego, CA, USA).

## Conflict of Interest

The authors declare no conflict of interest.

## Supporting information

Supplementary Material

## Data Availability

The data that support the findings of this study are available from the corresponding author upon reasonable request.
