# Supplementary material for: A Label‐Free Multitechnique Approach to Characterize the Interaction of Bioactive Compounds with Biomimetic Interfaces
Source: Small Sci. 2024 Mar 1;4(4):2300271. doi: 10.1002/smsc.202300271 (PMC11935184; doi:10.1002/smsc.202300271)
Supplement: Supplementary file 1 — Supplementary Material [file SMSC-4-2300271-s001.pdf]

Supporting Information

A label-free multitechnique approach to characterize the interaction of bioactive compounds with biomimetic interfaces

Eduarda Fernandes, Rui R. Costa, Raúl Machado, Rui L. Reis, Iva Pashkuleva and Marlene Lúcio**

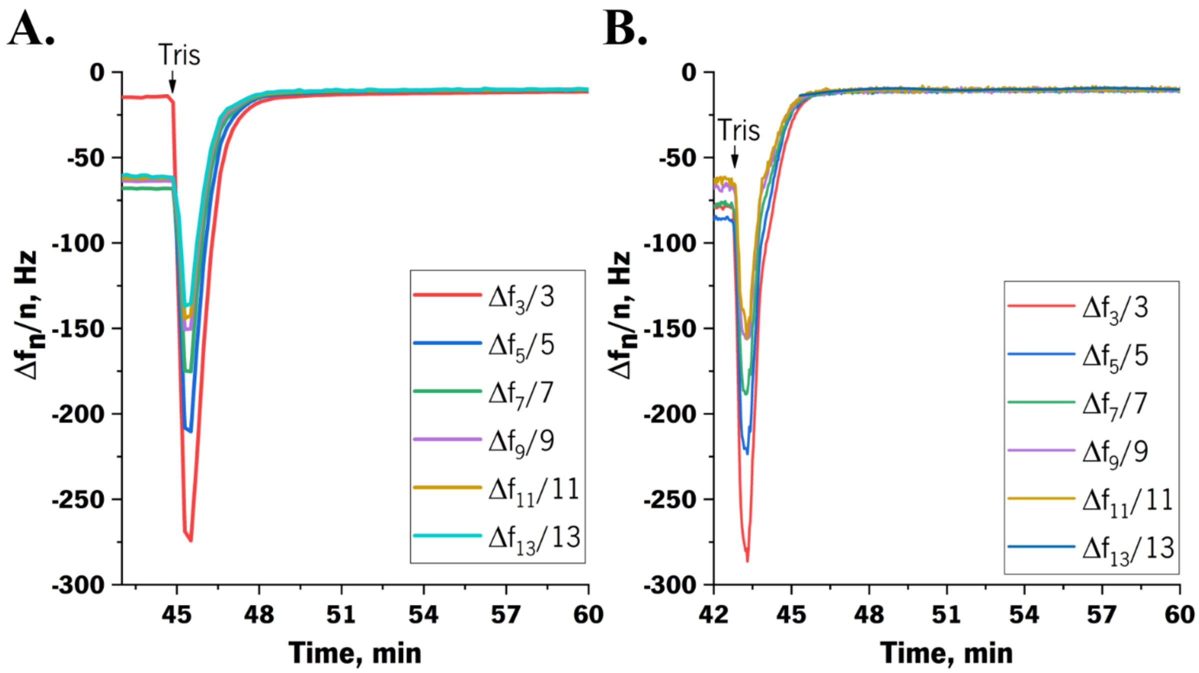

Figure S1. Variations of frequency (Δf) for all overtones (n=3–13) during the formation of SLBs of DPPC (B) and DPPC:Chol(2:1). The arrow indicates exchange of isopropanol by Tris, which is the final step of the SALB process. Time is scaled from the original graphs (Figure 2). All frequencies overlap by the end of SLB formation.

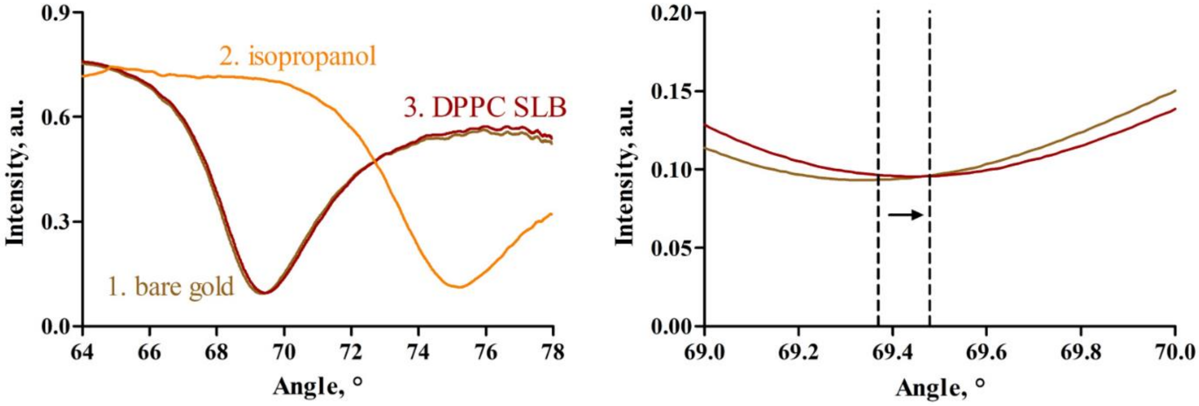

Figure S2. Characteristic reflectivity curves of the 670 nm laser of DPPC bilayers. Curves for the initial unmodified gold (1), isopropanol (for baseline of solvent exchange step, 2) and SLB (3) are represented. The right inset shows the curves zoomed in to show peak shifts.

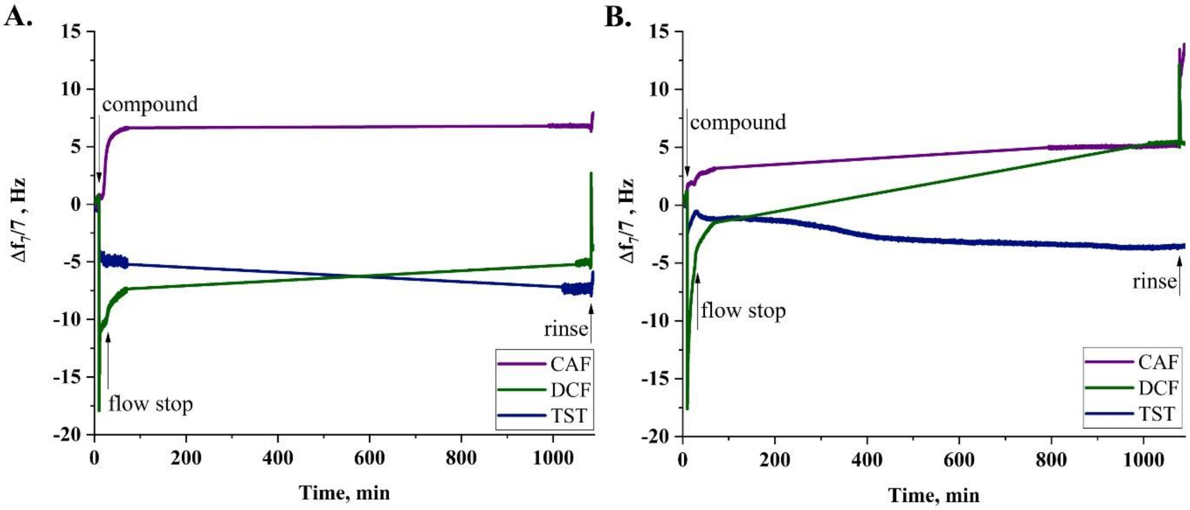

Figure S3. Full time range of frequency variation upon the interaction of CAF (purple), DCF (green) and TST (blue), with (A) DPPC and (B) DPPC:Chol(2:1) SLBs. The time-points of compound injection, flow stop, and final rinsing are indicated by the arrows.

Table S1. IR band assignments for single lipid DPPC and binary mixture DPPC:Chol(2:1).

Sample	Frequency (cm^{-1})	Assignment

	2958 / 2960	$v_{as}(CH_3)$
	2919 / 2921	$v_{as}(CH_2)$
	2874 / 2874	$v_s(CH_3)$
	2851 / 2852	$v_s(CH_2)$
	1735/1732	v(C=O)
**DPPC/DPPC:Chol(2:1)**	1487/1491	$\delta_{as}(N\text{-}CH_3)$
	1469/1468	$\delta(CH_2)$
	1378/1384	$\delta(CH_3)$
	1244/1244	$v_{as}(PO_2^-)$
	1092/1094	$v_s(PO_2^-)$
	1177/1179	v_{as}(C-O)

Vibrational modes: as = asymmetric; s = symmetric; ν = stretching; δ = bending; ω=wagging.

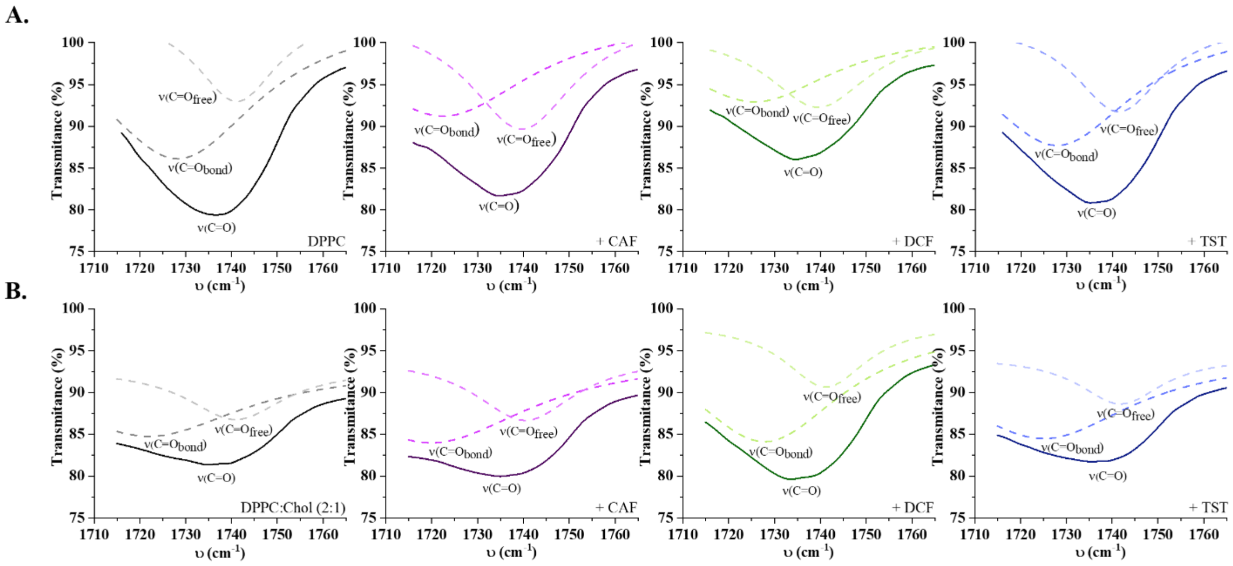

Figure S4. ν(C=O) band (solid lines) with corresponding Lorentzian deconvolution (dashed lines) in its hydrogen-free or -bond conformers of the biomimetic membrane models of: (A) DPPC or (B) DPPC:Chol(2:1) in the absence and presence of CAF (purple), DCF (green) and TST (blue).

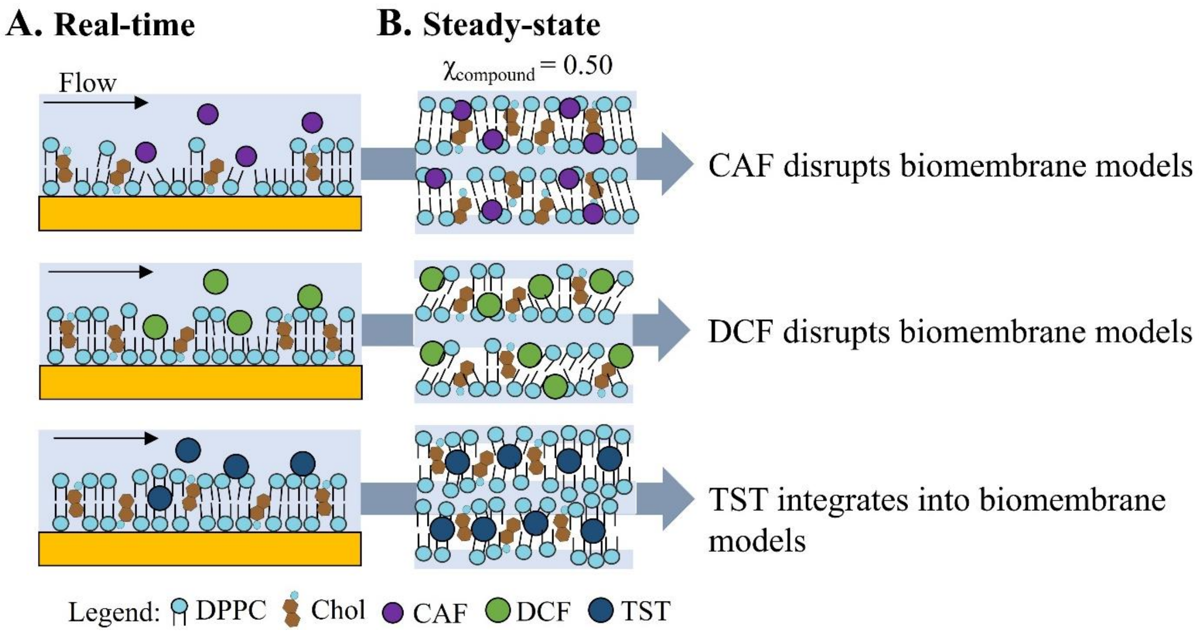

Figure S5. Schematic depiction of the interaction of the bioactives (CAF, DCF and TS) in the DPPC:Chol (2:1) SLBs and multi-stacked bilayers as membrane models monitored by real-time (A) and steady-state (B) techniques.
